# Supplementary material for: Future of Dutch NGS-Based Newborn Screening: Exploring the Technical Possibilities and Assessment of a Variant Classification Strategy
Source: Int J Neonatal Screen. 2024 Mar 7;10(1):20. doi: 10.3390/ijns10010020 (PMC10971161; doi:10.3390/ijns10010020)
Supplement: Supplementary file 1 [file IJNS-10-00020-s001.zip › 20231205 Supplementary files_definitief_nieuwerefs.pdf]

Future of Dutch NGS-based newborn screening: exploring the technical possibilities and assessment of a variant classification strategy

Authors: Gea Kiewiet[1*], Dineke Westra[2*], Eddy N. de Boer[1*], Emma van Berkel[2], Tom.G.J. Hofste[2], Martine van Zweeden[2], Ronny C. Derks[2], Nico F.A. Leijsten[2], Martina H.A. Ruiterkamp-Versteeg[2], Bart Charbon[1], Lennart Johansson[1], Janneke Bos-Kruizinga[1], Inge J. Veenstra[1], Monique G.M. de Sain-van der Velden[3], Els Voorhoeve[4], M. Rebecca Heiner-Fokkema[5], Francjan van Spronsen[6], Birgit Sikkema-Raddatz[1**], Marcel Nelen[2,7**]

Institutional affiliations:

[1]Department of Genetics, University Medical Center Groningen, University of Groningen, Groningen, Netherlands

[2]Department of Human Genetics, Radboud university medical center, Nijmegen, The Netherlands

[3]Section Metabolic Diagnostics, Department of Genetics, University Medical Center Utrecht, The Netherlands

[4]Center for Health Protection, National Institute for Public Health and the Environment, Bilthoven, The Netherlands

[5]Laboratory of Metabolic Diseases, Department of Laboratory Medicine, University Medical Center Groningen, University of Groningen, Groningen, The Netherlands

[6]Division of Metabolic Diseases, Beatrix Children's Hospital, University Medical Center Groningen, University of Groningen, Groningen, Netherlands

[7]Department of Genetics, University Medical Center Utrecht, Utrecht, The Netherlands.

* *Contributed equally as co-first authors*

** *Contributed equally as co-senior authors*

METHODS

Dried blood spots and DNA extraction

Fifty dried blood spots (DBS) from patients with an inherited metabolic disorder (IMD) associated with one of the selected 100 genes contained either capillary blood or Li-heparine anticoagulated blood. For the control samples, 75–80 µL of EDTA-anticoagulated blood were drawn on two half-inch (13 mm) circles of a DBS-card (Eastern Business Forms, Mauldin, SC, USA) and air dried for at least 3 hours at room temperature. DNA was extracted in two runs using half a blood spot for each DNA extraction. Punches were taken using a 6 mm manual puncher. The punches were washed with milliQ, after which TE buffer and proteinase K were added and lysis was done at 56°C for 2 hours. Next, automated DNA isolation was

performed on a Nimbus Presto robot (Hamilton, Manchester, ME, USA) using the Mag-Bind® Blood & Tissue DNA HDQ kit following the manufacturer's instructions (Omega Biotek, Norcross, GA, USA). Extracted DNA was quantified using a dropsense96 UV/Vis spectrophotometer (TRINEAN NV/SA, Gentbrugge, Belgium).
For the healthy individual cohort, DNA was isolated from EDTA whole blood samples on a chemagicSTAR (Hamilton Robotics, Reno, NV, USA).

Design of the targeted panel

A custom Sureselect capture panel was designed using the web-based design program SureDesign (Agilent, Santa Clara, California, USA). The panel includes all coding and non-coding exons and UTRs of the genes of interest (Table E1), as present in the RefSeq and Ensemble databases, together with some specific intronic regions that were considered clinically relevant. Probes were designed based on the GRCh37/hg19 build of the human genome, with 2x tiling frequency and boosted for optimized performance. By employing less stringent specificity settings for the remaining gaps in previous design rounds, multiple probe groups were eventually generated and merged to create a final design with maximum coverage. The performance of the panel was confirmed in a standard experiment on a set of reference DNA samples before its use in experiments with DBS samples.

Preparation of samples and sequencing targeted panel

For tNGS, libraries were automatically prepared following the SureSelect XT low input target enrichment system protocol on the Bravo NGS workstation (Agilent Technologies, Santa Clara, CA, USA). We used 100 ng DNA as input material. The intermediate products and library were qualified and quantified using a tapestation 2200 bioanalyzer (D1000 screentapes and reagents) (Agilent Technologies, Santa Clara, CA, USA). When library preparation failed, the procedure was repeated once for each sample, if enough DNA was available. The sequence procedure was executed on a NextSeq500 sequencer (Illumina, San Diego, USA). Read alignment and single nucleotide variant (SNV) and indel calling was performed using the MOLGENIS NGS_DNA pipeline version 3.6.0 (https://github.com/molgenis/NGS_DNA), using the Burrows-Wheeler Aligner (BWA) for alignment [1] against human reference genome build b37 as released by the 1000 Genomes Project [2]. The Genome Analysis Toolkit [3], version 3.7, was used for SNV and indel calling, annotation and coverage calculations. To call copy number variations (CNVs), we used CoNVaDING 1.2.1 [4] with the default settings. To create the control group, a preliminary run was performed including all DBS samples as control group. For the final analysis, samples having one or more calls on the shortlist were excluded as control. Using this control group, all DBS samples were examined for CNVs using the reduced control

group. CNVs on the final list were regarded as a CNV call. For data analysis, vcf files were uploaded into the Alissa clinical informatics platform (Agilent technologies). Analysis of sequence variants in the 100 selected genes (SNVs and small indels) was performed using Alissa Interpret software (Agilent Technologies).

Preparation of samples and sequencing WES

WES was performed similarly to previous reports, with some modifications [5]. In brief, DBS DNA samples were processed using the Human Core Exome Kit and extended RefSeq targets (Twist Biosciences, South San Francisco, CA, USA); for the background cohort, the exome was captured with the Agilent SureSelect v4/v5 kit (Agilent Technologies, Santa Clara, CA, USA). Libraries were prepared according to the manufacturers' protocols. All DNA samples were sheared using a Covaris R230 ultrasonicator (Covaris, Woburn, MA, USA), equimolar pooled (anticipating 100-fold coverage) and sequenced using 2 × 150bp paired-end sequencing on a HiSeq or Novaseq 6000 instrument (Illumina, San Diego, CA, USA). Downstream processing was performed using an automated data analysis pipeline that includes BWA mapping (human reference genome (GRCh37/hg19)), Genome Analysis Toolkit (GATK) calling for Single Nucleotide Variants (SNVs), CoNIFER and ExomeDepth calling for Copy Number Variants (CNVs) for bloodspot DNA, and custom-made annotation [6]. An in-silico gene-panel analysis was conducted encompassing the list of 100 established genes (Table E1).

Preparation of samples and sequencing WGS

WGS was performed as described by the manufacturer (Illumina, San Diego, CA, USA). In brief, 100 ng DNA was used for manual library preparation using the Illumina DNA PCR-free library prep protocol. Insert size was set at an average of 450 bp by shearing DNA using a Covaris R230 ultrasonicator (Covaris, Woburn, MA, USA). Samples were equimolarly pooled followed by 2 × 150 bp paired-end sequencing using two Illumina S4 flowcells (kindly provided by Illumina) on a NovaSeq6000™ instrument, anticipating a genome-wide coverage of 30-fold. FASTQ files were processed through our in-house developed GS-pipeline. Reads were mapped to the human reference genome (GRCh38/hg38) using BWA (v.0.78). Variant calling was performed per variant type using dedicated tools to optimize sensitivity, followed by variant annotation to facilitate variant interpretation. Similar in-silico gene-panel analysis was conducted encompassing the list of 100 established genes (Table E1).

Definitions of TP/TN/FP/FN in the (L)P filter strategy

In the strict filtering strategy reporting only pathogenic (P) and likely pathogenic (LP) variants, a sample was regarded **true positive (TP)** if one P or LP variant was detected with a variant

allele frequency (VAF) of 40-60% in an autosomal dominant (AD) gene or with a VAF of >90% in an autosomal recessive (AR) gene or X-linked recessive (XL) gene, or if two LP or P variants were detected with a VAF of 40-60% in an AR gene in an IMD-positive sample. A **false positive (FP)** was when these possibilities were detected in a control sample or in a gene other than the known affected gene in an IMD-positive sample. In the BC, all individual with a (L)P variant are considered FP, as we assume they will not have one of the target diseases*.* A sample was regarded **false negative (FN)** when the known (LP or P) variant was not detected in the AD or XL gene or the two known LP or P variants were not detected in the AR gene in an IMD-positive sample. **True negative (TN)** samples were control samples in which no LP or P variant detected with a VAF of 40-60% in an AD or with a VAF of >90% in an AR or XL gene or in which two LP or P variants were detected with a VAF of 40-60% in an AR gene.

Table S1: Genes and associated disorders included in gene panel

Gene	Genomic location (GRCh37)	Disorder	OMIM disease #	Mode of inheritance	Disease in Dutch NBS?
ABCD1	chrX:152,990,323-153,010,216 forward strand	X-linked adrenoleukodystrophy	300100	XLR	No
ACADM	chr1:76,190,036-76,253,260 forward strand	Medium-chain acyl-CoA dehydrogenase deficiency	201450	AR	Yes
ACADVL	chr17:7,120,444-7,128,592 forward strand	Very long-chain acyl-CoA dehydrogenase deficiency	201475	AR	Yes
ACAT1	chr11:107,992,243-108,018,503 forward strand	Mitochondrial acetoacetyl-CoA thiolase deficiency	203750	AR	No
ADA	chr20:43,248,163-43,280,874 reverse strand	Severe combined immunodeficiency (due to adenosine deaminase deficiency)	102700	AR	Yes
AGL	chr1:100,315,640-100,389,579 forward strand	Glycogen storage disease type 3 (due to glycogen debranching enzyme deficiency)	232400	AR	No
AGXT	chr2:241,807,896-241,819,919 forward strand	Primary hyperoxaluria type 1 (due to alanine-glyoxylate aminotransferase deficiency)	259900	AR	No
AHCY	chr20:32,868,074-32,899,608 reverse strand	S-adenosylhomocysteine hydrolase deficiency	613752	AR	No
AKR1D1	chr7:137,687,070-137,802,732 forward strand	Congenital bile acid synthesis defect type 2 (due to Δ4-3-oxosteroid 5β-reductase deficiency)	235555	AR	No
AKT2	chr19:40,736,224-40,791,443 reverse strand	Hypoinsulinemic hypoglycemia with hemihypertrophy (due to AKT2 superactivity)	240900	AD	No
ALDH7A1	chr5:125,877,533-125,931,110 reverse strand	Pyridoxine-dependent epilepsy (due to α-aminoadipic semialdehyde dehydrogenase deficiency)	266100	AR	No
ALDOB	chr9:104,182,860-104,198,105 reverse strand	Hereditary fructose intolerance (due to aldolase B deficiency)	229600	AR	No
AMN	chr14:103,388,993-103,399,933 forward strand	Imerslund-Gräsbeck syndrome, Norwegian type (due to amnionless deficiency)	618882	AR	No
APOC2	chr19:45,449,243-45,452,822 forward strand	Familial apolipoprotein C2 deficiency	207750	AR	No
APOE	chr19:45,409,011-45,412,650 forward strand	Dysbetalipoproteinemia / Hyperlipoproteinemia type 3 (due to apolipoprotein E deficiency)	617347	AR	No
ARG1	chr6:131,894,284-131,905,472 forward strand	Argininemia (due to arginase deficiency)	207800	AR	No
ARSA	chr22:51,061,182-51,066,607 reverse strand	Metachromatic leukodystrophy (due to arylsulfatase A deficiency)	250100	AR	No
ASL	chr7:65,540,785-65,558,545 forward strand	Argininosuccinic aciduria (due to argininosuccinate lyase deficiency)	207900	AR	No
ASS1	chr9:133,320,316-133,376,661 forward strand	Citrullinemia type 1 (due to argininosuccinate synthetase deficiency)	215700	AR	No
ATP7B	chr13:52,506,809-52,585,630 reverse strand	Wilson disease (due to copper-transporting ATPase β subunit deficiency)	277900	AR	No
BAAT	chr 9:104,122,699-104,145,801 reverse strand	Bile acid conjugation defect 1 (due to bile acid-CoA:amino acid N-acyltransferase deficiency)	619232	AR	No
BCKDHA	chr19:41,884,215-41,930,910 forward strand	Maple syrup urine disease type 1a (due to branched-chain ketoacid dehydrogenase E1α deficiency)	248600	AR	Yes
BCKDHB	chr6:80,816,364-81,055,987 forward strand	Maple syrup urine disease type 1b (due to branched-chain ketoacid dehydrogenase E1β deficiency)	248600	AR	No
BCKDK	chr16:31,117,428-31,124,110 forward strand	Autism-epilepsy syndrome (due to branched-chain ketoacid dehydrogenase kinase deficiency)	614923	AR	No

Gene	Genomic location (GRCh37)	Disorder	OMIM disease #	Mode of inheritance	Disease in Dutch NBS?
BTD	chr3:15,642,848-15,687,329 forward strand	Biotinidase deficiency	253260	AR	Yes
CA5A	chr16:87,921,625-87,970,135 reverse strand	Hyperammonemic encephalopathy (due to carbonic anhydrase VA deficiency)	615751	AR	No
CAD	chr2:27,440,258-27,466,811 forward strand	Congenital defect of glycosylation (CDG) type 1z (due to CAD trifunctional protein deficiency)	616457	AR	No
CBS	chr21:44,473,301-44,497,053 reverse strand	Classic homocystinuria (due to cystathionine β-synthase deficiency)	236200	AR	No
CPS1	chr2:211,342,406-211,543,831 forward strand	Carbamoyl phosphate synthetase 1 deficiency	237300	AR	No
CPT1A	chr11:68,522,088-68,611,878 reverse strand	Carnitine palmitoyltransferase 1a deficiency	255120	AR	Yes
CPT2	chr1:53,662,101-53,679,869 forward strand	Carnitine palmitoyltransferase 2 deficiency, infantile;<br>Carnitine palmitoyltransferase 2 deficiency, lethal neonatal	600649;<br>608836	AR	No
CTNS	chr17:3,539,762-3,564,836 forward strand	Cystinosis	219800	AR	No
CTPS1	chr1:41,445,007-41,478,235 forward strand	Severe combined immunodeficiency (due to CTP synthase 1 deficiency)	615897	AR	Yes
CYP27A1	chr2:219,646,472-219,680,016 forward strand	Cerebrotendinous xanthomatosis (due to sterol 27-hydroxylase deficiency)	213700	AR	No
DBT	chr1:100,652,475-100,715,390 reverse strand	Maple syrup urine disease type 2 (due to dihydrolipoyl transacylase deficiency)	248600	AR	No
DNAJC12	chr10:69,556,427-69,597,924 reverse strand	Hyperphenylalaninemia (due to DNAJC12 deficiency)	617384	AR	No
ETFA	chr15:76,507,696-76,603,813 reverse strand	Glutaric acidemia type 2a (due to electron transfer flavoprotein α subunit deficiency)	231680	AR	No
ETFB	chr19:51,848,423-51,869,672 reverse strand	Glutaric acidemia type 2b (due to electron transfer flavoprotein β subunit deficiency)	231680	AR	No
ETFDH	chr4:159,593,277-159,630,775 forward strand	Glutaric acidemia type 2c (due to electron transfer flavoprotein dehydrogenase deficiency)	231680	AR	No
FAH	chr15:80,444,832-80,479,288 forward strand	Tyrosinemia type 1 (due to fumarylacetoacetase deficiency)	276700	AR	Yes
FBP1	chr9:97,365,415-97,402,531 reverse strand	Fructose-1,6-bisphosphatase deficiency	229700	AR	No
FOLR1	chr11:71,900,602-71,907,345 forward strand	Neurodegeneration (due to cerebral folate receptor α deficiency)	613068	AR	No
G6PC	chr17:41,052,814-41,065,386 forward strand	Glycogen storage disease type 1a (due to glucose-6-phosphatase deficiency)	232200	AR	No
GALK1	chr17:73,747,675-73,761,792 reverse strand	Galactokinase deficiency	230200	AR	Yes
GALT	chr9:34,638,130-34,651,032 forward strand	Classic galactosemia (due to galactose-1-phosphate uridylyltransferase deficiency)	230400	AR	Yes
GAMT	chr19:1,397,091-1,401,569 reverse strand	Guanidinoacetate methyltransferase deficiency	612736	AR	No
GATM	chr15:45,653,322-45,694,525 reverse strand	Cerebral creatine deficiency syndrome 3 (due to arginine:glycine amidinotransferase deficiency)	612718	AR	No
GBA	Chr1:155,204,243-155,214,490 reverse strand	Gaucher disease type III (due to nonlysosomal glucosylceramidase deficiency)	231000	AR	No
GCDH	chr19:13,001,840-13,025,021 forward strand	Glutaric acidemia type 1 (due to glutaryl-CoA dehydrogenase deficiency)	231670	AR	Yes
GCH1	chr14:55,308,726-55,369,570 reverse strand	Hyperphenylalaninemia , BH4-deficient, B (due to GTP cyclohydrolase 1 deficiency)	233910	AR	No
GCK	chr7:44,183,872-44,237,769 reverse strand	Hyperinsulinemic hypoglycemia (due to glucokinase deficiency);<br>Diabetes mellitus, permanent neonatal 1 (due to glucokinase deficiency)	602485;<br>606176	AD;<br>AR	No

Gene	Genomic location (GRCh37)	Disorder	OMIM disease #	Mode of inheritance	Disease in Dutch NBS?
GLUD1	chr10:88,810,243-88,854,623 reverse strand	Hyperinsulinism-hyperammonemia syndrome (due to glutamate dehydrogenase superactivity)	606762	AD	No
GPIHBP1	chr8:144,295,068-144,299,044 forward strand	GPIHBP1 deficiency	615947	AR	No
GYS2	chr12:21,689,123-21,757,781 reverse strand	Glycogen storage disease type 0a (due to hepatic glycogen synthase deficiency)	240600	AR	No
HADHA	chr2:26,413,504-26,467,594 reverse strand	Long-chain 3-hydroxyl-CoA dehydrogenase deficiency (due to trifunctional protein α subunit deficiency)	609016	AR	Yes
HJV	chr1:145,413,095-145,417,545 forward strand	Hemochromatosis type 2 (due to hHemojuvelin deficiency)	602390	AR	No
HLCS	chr21:38,123,189-38,362,536 reverse strand	Holocarboxylase synthetase deficiency	253270	AR	Yes
HMGCL	chr1:24,128,375-24,165,110 reverse strand	3-hydroxy-3-methylglutaryl-CoA lyase deficiency	246450	AR	Yes
HMGCS2	chr1:120,290,619-120,311,528 reverse strand	Mitochondrial 3-hydroxy-3-methylglutaryl-CoA synthase deficiency	605911	AR	No
HSD3B7	chr16:30,996,519-31,000,473 forward strand	Congenital bile acid synthesis defect type 1 (due to 3β-Hydroxy-Δ5-C27-steroid oxidoreductase deficiency)	607765	AR	No
IDUA	chr4:980,785-998,316 forward strand	Hurler syndrome (due to α-iduronidase deficiency)	607015	AR	Yes
IVD	chr15:40,697,686-40,728,146 forward strand	Isovaleric acidemia (due to isovaleryl-CoA dehydrogenase deficiency)	243500	AR	Yes
LPL	chr8:19,759,228-19,824,769 forward strand	Lipoprotein lipase deficiency	238600	AR	No
MCCC1	chr3:182,733,006-182,833,863 reverse strand	3-methylcrotonylglycinuria type 1 (due to 3-Methylcrotonyl-CoA carboxylase 1 deficiency)	210200	AR	Yes
MCCC2	chr5:70,883,115-70,954,531 forward strand	3-methylcrotonylglycinuria type 2 (due to 3-Methylcrotonyl-CoA carboxylase 2 deficiency)	210210	AR	Yes
MCEE	chr2:71,336,814-71,357,369 reverse strand	Methylmalonic aciduria (due to methylmalonyl-CoA epimerase deficiency)	251120	AR	Yes
MMAA	chr4:146,539,415-146,581,187 forward strand	Methylmalonic aciduria, cblA type	251100	AR	No
MMACHC	chr1:45,965,725-45,976,739 forward strand	Methylmalonic aciduria with homocystinuria, clbC type	277400	AR	No
MMADHC	chr21:44,473,301-44,497,053 reverse strand	Homocystinuria without methylmalonic aciduria, cblD type	277410	AR	No
MMUT	chr6:49,398,073-49,430,904 reverse strand	Methylmalonic aciduria (due to methylmalonyl-CoA mutase deficiency)	251000	AR	Yes
NAGS	chr17:42,081,914-42,086,431 forward strand	Hyperammonemia (due to N-acetylglutamate synthase deficiency)	237310	AR	No
OAT	chr10:126,085,872-126,107,545 reverse strand	Gyrate atrophy of choroid and retina (due to ornithine aminotransferase deficiency)	258870	AR	No
OTC	chrX:38,211,798-38,280,703 forward strand	Ornithine transcarbamylase deficiency	311250	XLR	No
OXCT1	chr5:41,730,167-41,870,621 reverse strand	Succinyl-CoA:3-oxoacid-CoA transferase (SCOT) deficiency	245050	AR	No
PAH	chr12:103,230,663-103,352,188 reverse strand	Phenylketonuria (due to phenylalanine hydroxylase deficiency)	261600	AR	Yes
PCBD1	chr10:72,642,037-72,648,541 reverse strand	Pterin-4-α-carbinolamine dehydratase deficiency	264070	AR	No
PCCA	chr13:100,741,269-101,182,686 forward strand	Propionic acidemia (due to propionyl-CoA carboxylase α subunit deficiency)	606054	AR	Yes
PCCB	chr3:135,969,148-136,056,738 forward strand	Propionic acidemia (due to propionyl-CoA carboxylase β subunit deficiency)	606054	AR	Yes
PGM1	chr1:64,058,947-64,125,916 forward strand	Congenital defect of glycosylation (CDG) type 1t (due to phosphoglucomutase 1	614921	AR	No

Gene	Genomic location (GRCh37)	Disorder	OMIM disease #	Mode of inheritance	Disease in Dutch NBS?
		deficiency)			
PNP	chr14:20,937,113-20,945,253 forward strand	Purine nucleoside phosphorylase deficiency	613179	AR	No
PTS	chr11:112,097,088-112,140,678 forward strand	6-pyruvoyl-tetrahydropterin synthase deficiency	261640	AR	No
QDPR	chr4:17,461,884-17,513,857 reverse strand	Dihydropteridine reductase deficiency	261630	AR	No
SI	chr3:164,696,686-164,796,283 reverse strand	Congenital sucrase-isomaltase deficiency	222900	AR	No
SLC19A3	chr2:228,549,926-228,582,728 reverse strand	Biotin-thiamine-responsive basal ganglia disease (due to thiamine transporter 2 deficiency)	607483	AR	No
SLC22A5	chr5:131,705,444-131,731,306 forward strand	Primary carnitine deficiency	212140	AR	No
SLC25A15	chr13:41,363,548-41,384,247 forward strand	Hyperornithinemia-hyperammonemia-homocitrullinuria syndrome (due to Mitochondrial ornithine transporter deficiency)	238970	AR	No
SLC25A20	chr3:48,894,369-48,936,426 reverse strand	Carnitine-acylcarnitine translocase deficiency	212138	AR	No
SLC2A1	chr1:43,391,052-43,424,530 reverse strand	GLUT1 deficiency	606777	AD	No
SLC2A2	chr3:170,714,137-170,744,539 reverse strand	Fanconi-Bickel syndrome (due to glucose transporter 2 deficiency)	227810	AR	No
SLC37A4	chr11:118,894,824-118,901,616 reverse strand	Glycogen storage disease type 1b (due to glucose-6-phosphate transporter deficiency)	232220	AR	No
SLC40A1	chr2:190,425,305-190,448,484 reverse strand	Hereditary hemochromatosis type 4 (due to ferroportin deficiency)	606069	AD	No
SLC46A1	Chr17:26,721,661-26,734,215 reverse strand	Hereditary folate malabsorption (due to proton-coupled folate transporter deficiency)	229050	AR	No
SLC52A2	chr8:145,577,795-145,584,932 forward strand	Brown-Vialetto van Laere syndrome type 2 (due to riboflavin transporter 3 deficiency)	614707	AR	No
SLC52A3	chr20:740,724-749,131 reverse strand	Brown-Vialetto van Laere syndrome type 1 (due to riboflavin transporter 2 deficiency)	211530	AR	No
SLC5A1	chr22:32,439,019-32,509,016 forward strand	Glucose-galactose malabsorption (due to intestinal sodium-glucose cotransporter 1 deficiency)	606824	AR	No
TAT	chr16:71,599,563-71,611,033 reverse strand	Tyrosinemia type 2 (due to tyrosine aminotransferase deficiency)	276600	AR	No
TCN2	chr22:31,002,825-31,023,265 forward strand	Transcobalamin 2 deficiency	275350	AR	No
TH	chr11:2,185,159-2,193,107 reverse strand	Segawa syndrome (due to tyrosine hydroxylase deficiency)	605407	AR	No
TPK1	chr7:144,149,034-144,533,488 reverse strand	Episodic encephalopathy (due to thiamine pyrophosphokinase deficiency)	614458	AR	No
TTPA	chr8:63,961,112-63,998,612 reverse strand	Ataxia with isolated vitamin E deficiency (due to α-tocopherol transfer protein deficiency)	277460	AR	No

Table S2: Identified homozygous pathogenic variants in three individuals in the background population that would be reported in a NBS setting.

ID	Variant	Comment
S2209	*ACADVL* Chr17(GRCh37) :g.7125591T>C NM_000018.4 :c.848T>C p.(Val283Ala) ; hom.	VLCAD deficiency; variant associated with mild phenotype, (late onset, one or few episodes of metabolic decompensation) [7].
S4220	*CPT1A* Chr11(GRCh37):g.68548130G>A NM_001876.4:c.1436C>T p.(Pro479Leu); hom.	CPT I deficiency; variant associated with variable phenotype (healthy to severe) [8].
S2906	*MCCC2* Chr5(GRCh37):g.70945016A>G NM_022132.5:c.1309A>G p.(Ile437fs); hom.	3-methylcrotonyl-CoA carboxylase 2 deficiency; variant associated with variable phenotype with asymptomatic homozygous siblings described [9].

Table S3: Identified combinations of a (L)P variant and VUS in four individuals in the background cohort that might need to be reported in a NBS setting.

ID	Variant	Comment
S4422	*AGL* Chr1(GRCh37):g.100361878del NM_000642.3:c.3299del p.(Gly1100fs); het.	Pathogenic: frameshift resulting in NMD
	AGL Chr1(GRCh37):g.100377978A>C NM_000642.3:c.3854A>C p.(Glu1285Ala); het.	VUS: 3/4 deleterious *in silico* predictions
S4007	*CBS* Chr21(GRCh37):g.44479080T>G NM_000071.3:c.1224-2A>C p.Trp408_Gly453del; het.	Pathogenic: in-frame deletion of exon 12[10]; no activity detected[11].
	CBS Chr21(GRCh37):g.44480591G>A NM_000071.3:c.1105C>T p.(Arg369Cys); het.	VUS: Yeast similar to WT[12]; 34% and 54% activity in E. coli and CHO-K1[13].
S255	*GBA* Chr1(GRCh37):g.155205043A>G NM_000157.4:c.1448T>C p.(Leu483Pro); het.	Pathogenic: most common variant in Gaucher's disease; reduced half-life, because protein gets degraded[14].
	GBA Chr1(GRCh37):g.155206036C>T NM_000157.4:c.1224G>A p.(Thr408=); het.	VUS: Splicing defect predicted in Alamut; in-frame PD; not in SpliceAI; found in Parkinson's disease[15].
S4762	*SI* Chr3(GRCh37):g.164741534A>G NM_001041.4:c.2923T>C p.(Tyr975His); het.	VUS: described in a low sucrase patient[16].
	SI Chr3(GRCh37):g.164735598_164735599del NM_001041.4:c.3586_3587del p.(Met1196fs); het.	Likely pathogenic: frameshift resulting in NMD; Classified as LP by ACMG guideline in literature[17].

Table S4. List of heterozygous (likely) pathogenic variants in autosomal recessive genes, indicating carriership, detected with one or more NGS techniques.

Sample ID	Heterozygous variant	Detected with
2	*BTD* Chr3(GRCh37):g.15686731A>C NM_001370658.1:c.1308A>C p.(Gln436His)	tNGS/WES/WGS
11/27/41	*LPL* Chr8(GRCh37):g.19811685C>G NM_000237.3:c.596C>G p.(Ser199Cys)	tNGS
17	*ARSA* Chr22(GRCh37):g.51063820G>A NM_000487.6:c.1283C>T p.(Pro428Leu)	tNGS/WES/WGS
18	*SI* Chr3(GRCh37):g.164739053C>T NM_001041.4:c.3218G>A p.(Gly1073Asp)	tNGS
32	*MCCC1* Chr3(GRCh37):g.182759428_182759429del NM_020166.4:c.1193_1194del p.(Val398fs)	WES/WGS
42	*MCCC2* Chr5(GRCh37):g.70936845G>A NM_022132.5:c.1015G>A p.(Val339Met)	tNGS
43	*SI* Chr3(GRCh37):g.164764786A>C NM_001041.4:c.1730T>G p.(Val577Gly)	tNGS
45	*TTPA* Chr8(GRCh37):g.63978594C>T NM_000370.3:c.421G>A p.(Glu141Lys)	WES/WGS
46	*TH* Chr11(GRCh37):g.2189135C>T NM_199292.1:c.698G>A p.(Arg233His)	tNGS
Negative control	*PCCB* Chr3(GRCh37):g.136035907G>A NM_001178014.1:c.1150+1G>A r.spl p.?	WES
Negative control	*MCEE* Chr2(GRCh37):g.71351575G>A NM_032601.4:c.139C>T p.(Arg47*)	tNGS/WES
Negative control	*ACADM* Chr1(GRCh37):g.76226933A>G NM_001286042.1:c.964A>G p.(Lys211Glu)	WES
Negative control	*ATP7B* Chr13(GRCh37)g.52524268C>T NM_000053.4(ATP7B):c.2605G>A p.(Gly869Arg)	tNGS/WES
Negative control	*PAH* Chr12(GRCh37):g.103246714G>A NM_000277.1:c.721C>T p.(Arg241Cys)	WES
Negative control	*GALT* Chr9(GRCh37):g.34648167A>G NM_000155.3:c.563A>G p.(Gln188Arg)	tNGS
Negative control	*PCCB* Chr3(GRCh37):g.135969390A>C NM_001178014.1:c.173A>C p.(Gln58Pro)	tNGS/WES
Negative control (2x)	*ALDOB* Chr9(GRCh37):g.104189856C>G NM_000035.3:c.448G>C p.(Ala150Pro)	tNGS/WES
Negative control (2x)	*APOC2* Chr19(GRCh37):g.45452024A>C NM_000483.5:c.122A>C p.(Lys41Thr)	tNGS
Negative control	*ACADM* Chr1(GRCh37):g.76226846A>G NM_000016.5:c.985A>G p.(Lys329Glu)	tNGS
Negative control	*PAH* Chr12(GRCh37):g.103246708G>A NM_000277.2:c.727C>T p.(Arg243*)	tNGS
Negative control (2x)	*SLC22A5* Chr5(GRCh37):g.131705516G>A NM_003060.3:c.-149G>A p.?	tNGS
Negative control	*ACADVL* Chr17(GRCh37):g.7125591T>C NM_000018.3:c.848T>C p.(Val283Ala)	tNGS
Negative control	*CBS* Chr21(GRCh37):g.44483184A>G NM_000071.2:c.833T>C p.(Ile278Thr)	tNGS

References Supplementary files

1. Li, H.; Durbin, R. Fast and Accurate Long-Read Alignment with Burrows–Wheeler Transform. *Bioinformatics* **2010,** *26*, 589-595**.**

2. Altshuler, D.L.; Durbin, R.M.; Abecasis, G.R.; Bentley, D.R.; Chakravarti, A.; Clark, A.G.; Collins, F.S.; de La Vega, F.M.; Donnelly, P.; Egholm, M.; et al. A Map of Human Genome Variation from Population Scale Sequencing. *Nature* **2010**, *467*, 1061, doi:10.1038/NATURE09534.

3. McKenna, A.; Hanna, M.; Banks, E.; Sivachenko, A.; Cibulskis, K.; Kernytsky, A.; Garimella, K.; Altshuler, D.; Gabriel, S.; Daly, M.; et al. The Genome Analysis Toolkit: A MapReduce Framework for Analyzing next-Generation DNA Sequencing Data. *Genome Res* **2010**, *20*, 1297–1303, doi:10.1101/GR.107524.110.

4. Johansson, L.F.; van Dijk, F.; de Boer, E.N.; van Dijk-Bos, K.K.; Jongbloed, J.D.H.; van der Hout, A.H.; Westers, H.; Sinke, R.J.; Swertz, M.A.; Sijmons, R.H.; et al. CoNVaDING: Single Exon Variation Detection in Targeted NGS Data. *Hum Mutat* **2016**, *37*, 457–464, doi:10.1002/HUMU.22969.

5. Deden, C.; Neveling, K.; Zafeiropopoulou, D.; Gilissen, C.; Pfundt, R.; Rinne, T.; de Leeuw, N.; Faas, B.; Gardeitchik, T.; Sallevelt, S.C.E.H.; et al. Rapid Whole Exome Sequencing in Pregnancies to Identify the Underlying Genetic Cause in Fetuses with Congenital Anomalies Detected by Ultrasound Imaging. *Prenat Diagn* **2020**, *40*, 972–983, doi:10.1002/PD.5717.

6. Lelieveld, S.H.; Reijnders, M.R.F.; Pfundt, R.; Yntema, H.G.; Kamsteeg, E.J.; De Vries, P.; De Vries, B.B.A.; Willemsen, M.H.; Kleefstra, T.; Löhner, K.; et al. Meta-Analysis of 2,104 Trios Provides Support for 10 New Genes for Intellectual Disability. *Nat Neurosci* **2016**, *19*, 1194–1196, doi:10.1038/NN.4352.

7. Spiekerkoetter, U.; Sun, B.; Zytkovicz, T.; Wanders, R.; Strauss, A.W.; Wendel, U. MS/MS-Based Newborn and Family Screening Detects Asymptomatic Patients with Very-Long-Chain Acyl-CoA Dehydrogenase Deficiency. *Journal of Pediatrics* **2003**, *143*, 335–342, doi:10.1067/S0022-3476(03)00292-0.

8. Greenberg, C.R.; Dilling, L.A.; Thompson, G.R.; Seargeant, L.E.; Haworth, J.C.; Phillips, S.; Chan, A.; Vallance, H.D.; Waters, P.J.; Sinclair, G.; et al. The Paradox of the Carnitine Palmitoyltransferase Type Ia P479L Variant in Canadian Aboriginal Populations. *Mol Genet Metab* **2009**, *96*, 201–207, doi:10.1016/J.YMGME.2008.12.018.

9. Mourmans, J.; Bakkeren, J.; de Jong, J.; Wevers, R.; van Diggelen, O.P.; Suormala, T.; Baumgartner, R.; Wendel, U. Isolated (Biotin-Resistant) 3-Methylcrotonyl-CoA Carboxylase Deficiency: Four Sibs Devoid of Pathology. *J Inherit Metab Dis* **1995**, *18*, 643–645, doi:10.1007/BF02436014.

10. Kozich, V.; Kraus, J.P. Screening for Mutations by Expression Patient CDNA Segments in E. Coli: Homocystinuria Due to Cystathionine Beta-Synthase Deficiency. *Hum Mutat* **1992**, *1*, 113–123.

11. Kopecká, J.; Krijt, J.; Raková, K.; Kožich, V. Restoring Assembly and Activity of Cystathionine β-Synthase Mutants by Ligands and Chemical Chaperones. *J Inherit Metab Dis* **2011**, *34*, 39–48, doi:10.1007/s10545-010-9087-5.

12. Mayfield, J.A.; Davies, M.W.; Dimster-Denk, D.; Pleskac, N.; McCarthy, S.; Boydston, E.A.; Fink, L.; Lin, X.X.; Narain, A.S.; Meighan, M.; et al. Surrogate Genetics and Metabolic Profiling for Characterization of Human Disease Alleles. *Genetics* **2012**, *190*, 1309–1323, doi:10.1534/genetics.111.137471.

13. Janošík, M.; Sokolová, J.; Janošíková, B.; Krijt, J.; Klatovská, V.; Kožich, V. Birth Prevalence of Homocystinuria in Central Europe: Frequency and Pathogenicity of Mutation c.1105C>T (p.R369C) in the Cystathionine Beta-Synthase Gene. *Journal of Pediatrics* **2009**, *154*, 431–437, doi:10.1016/j.jpeds.2008.09.015.

14. Lu, J.; Yang, C.; Chen, M.; Ye, D.Y.; Lonser, R.R.; Brady, R.O.; Zhuang, Z. Histone Deacetylase Inhibitors Prevent the Degradation and Restore the Activity of Glucocerebrosidase in Gaucher Disease. *Proc Natl Acad Sci U S A* **2011**, *108*, 21200–21205, doi:10.1073/pnas.1119181109.

15. den Heijer, J.M.; Cullen, V.C.; Quadri, M.; Schmitz, A.; Hilt, D.C.; Lansbury, P.; Berendse, H.W.; van de Berg, W.D.J.; de Bie, R.M.A.; Boertien, J.M.; et al. A Large-Scale Full GBA1 Gene Screening in Parkinson’s Disease in the Netherlands. *Movement Disorders* **2020**, *35*, 1667–1674, doi:10.1002/mds.28112.

16. Deb, C.; Campion, S.; Derrick, V.; Ruiz, V.; Abomoelak, B.; Avdella, A.; Zou, B.; Horvath, K.; Mehta, D.I. Sucrase-Isomaltase Gene Variants in Patients with Abnormal Sucrase Activity and Functional Gastrointestinal Disorders. *J Pediatr Gastroenterol Nutr* **2021**, *72*, 29–35, doi:10.1097/MPG.0000000000002852.

17. Capalbo, A.; Valero, R.A.; Jimenez-Almazan, J.; Pardo, P.M.; Fabiani, M.; Jiménez, D.; Simon, C.; Rodriguez, J.M. Optimizing Clinical Exome Design and Parallel Gene-Testing for Recessive Genetic Conditions in Preconception Carrier Screening: Translational Research Genomic Data from 14,125 Exomes. *PLoS Genet* **2019**, *15*, doi:10.1371/journal.pgen.1008409.
