# Supplementary material for: Future of Dutch NGS-Based Newborn Screening: Exploring the Technical Possibilities and Assessment of a Variant Classification Strategy
Source: Int J Neonatal Screen. 2024 Mar 7;10(1):20. doi: 10.3390/ijns10010020 (PMC10971161; doi:10.3390/ijns10010020)
Supplement: Supplementary file 1 [file IJNS-10-00020-s001.zip › supplementaryfile2.pdf]

Supplementary file 2:overview of genome positions that did not comply with quality requirements per method

tNGS

**Chr**	**Start**	**End**	**Gene**
1	24135767	24135960	HMGCL
1	24135964	24135964	HMGCL
1	41445871	41445871	CTPS1
1	41466475	41466494	CTPS1
1	41466497	41466497	CTPS1
1	45975488	45975488	MMACHC
1	45975490	45975631	MMACHC
1	45975740	45975741	MMACHC
1	45977851	45978113	MMACHC
1	53679365	53679529	CPT2
1	64120651	64120690	PGM1
1	64120693	64120695	PGM1
1	76190049	76190123	ACADM
1	76216469	76216470	ACADM
1	76216546	76216586	ACADM
1	76220608	76220634	ACADM
1	76220722	76220784	ACADM
1	76229263	76229374	ACADM
1	100654908	100654908	DBT
1	100657306	100657331	DBT
1	100659255	100659257	DBT
1	100659265	100659269	DBT
1	100659273	100659320	DBT
1	100659960	100660192	DBT
1	100662091	100662092	DBT
1	100665207	100665207	DBT
1	100665209	100665395	DBT
1	100665914	100666120	DBT
1	100684836	100684842	DBT
1	100684846	100684846	DBT
1	100684849	100685049	DBT
1	100685826	100685826	DBT
1	120296000	120296019	HMGCS2
2	26457949	26458109	HADHA
2	26458112	26458112	HADHA
2	27462862	27463038	CAD
2	27463040	27463041	CAD
2	71336804	71336837	MCEE
2	71343893	71343897	MCEE
2	211539006	211539136	CPS1
2	211539207	211539208	CPS1
2	228548610	228548612	SLC19A3
2	228548621	228548621	SLC19A3
2	228548624	228548714	SLC19A3
2	228548719	228548719	SLC19A3

2	228548723	228548723	SLC19A3
2	228549501	228549596	SLC19A3
2	228550644	228550828	SLC19A3
2	228550830	228550834	SLC19A3
2	228560801	228560801	SLC19A3
2	228566352	228566352	SLC19A3
2	228571305	228571317	SLC19A3
2	228571319	228571319	SLC19A3
2	228571331	228571370	SLC19A3
2	241819023	241819024	AGXT
2	241819030	241819143	AGXT
2	241819923	241819929	AGXT
3	15691945	15691946	BTD
3	15692380	15692492	BTD
3	15693693	15693693	BTD
3	15693696	15693707	BTD
3	15693709	15693709	BTD
3	15753330	15753385	BTD
3	15753388	15753392	BTD
3	15753396	15753397	BTD
3	135971230	135971241	PCCB
3	135978982	135979019	PCCB
3	135979022	135979023	PCCB
3	136053694	136053983	PCCB
3	136055803	136055806	PCCB
3	164696676	164696803	SI
3	164714561	164714570	SI
3	164751174	164751174	SI
3	170727737	170727755	SLC2A2
3	182833424	182833599	MCCC1
4	993598	993598	IDUA
4	993603	993624	IDUA
4	996955	997070	IDUA
4	17488508	17488508	QDPR
4	17513758	17513758	QDPR
4	159601032	159601263	ETFDH
4	159630172	159630193	ETFDH
4	159630196	159630196	ETFDH
4	159630198	159630198	ETFDH
4	159630201	159630202	ETFDH
4	159630206	159630206	ETFDH
4	159630425	159630428	ETFDH
4	159630432	159630630	ETFDH
5	41739828	41739833	OXCT1
5	70932287	70932548	MCCC2
5	70953392	70953392	MCCC2
5	70953456	70953505	MCCC2
5	125878955	125878955	ALDH7A1
5	125900040	125900056	ALDH7A1
5	125907052	125907054	ALDH7A1

5	125929033	125929041	ALDH7A1
5	131713420	131713421	SLC22A5
5	131715273	131715392	SLC22A5
5	131730053	131730053	SLC22A5
6	81053717	81053719	BCKDHB
6	81053784	81054305	BCKDHB
6	81055961	81055962	BCKDHB
6	81055966	81055972	BCKDHB
6	81055979	81055997	BCKDHB
7	44182802	44182820	GCK
7	44187412	44187442	GCK
7	137783050	137783066	AKR1D1
7	137802498	137802706	AKR1D1
7	144316264	144316275	TPK1
7	144316282	144316300	TPK1
8	63961102	63961159	TTPA
8	63972316	63972332	TTPA
8	63972336	63972344	TTPA
8	63972373	63972374	TTPA
8	63972377	63972384	TTPA
8	63972398	63972455	TTPA
8	63973233	63973235	TTPA
8	63978659	63978659	TTPA
8	144297961	144298018	GPIHBP1
9	35741307	35741502	GBA2
9	35749100	35749101	GBA2
9	35749132	35749146	GBA2
9	35749151	35749155	GBA2
9	35749202	35749235	GBA2
9	97365415	97365415	FBP1
9	97401801	97401810	FBP1
9	104147127	104147298	BAAT
9	133320084	133320189	ASS1
9	133320195	133320198	ASS1
9	133320205	133320207	ASS1
9	133320244	133320284	ASS1
9	133376649	133376671	ASS1
10	69587290	69587395	DNAJC12
10	69587397	69587397	DNAJC12
10	69587429	69587447	DNAJC12
10	88835782	88835790	GLUD1
10	126100238	126100240	OAT
10	126103628	126103651	OAT
11	2189922	2189966	TH
11	2191995	2192010	TH
11	68522950	68523502	CPT1A
11	68586145	68586170	CPT1A
11	68609233	68609298	CPT1A
11	68609306	68609309	CPT1A
11	68609368	68609409	CPT1A

11	107999758	107999938	ACAT1
11	108005143	108005185	ACAT1
11	112140594	112140594	PTS
11	118898436	118898436	SLC37A4
13	41363538	41363637	SLC25A15
13	41363731	41363734	SLC25A15
13	41363736	41363809	SLC25A15
13	41384345	41384504	SLC25A15
13	41385306	41385537	SLC25A15
13	100813228	100813229	PCCA
15	40711511	40712283	IVD
15	40712349	40712349	IVD
15	40713491	40713491	IVD
15	45665424	45665539	GATM
15	45670725	45670737	GATM
15	45670742	45670742	GATM
15	45670746	45670746	GATM
15	45670748	45670753	GATM
15	76518681	76518686	ETFA
15	76522835	76523000	ETFA
15	76576696	76576811	ETFA
15	76577136	76577278	ETFA
15	76602358	76602358	ETFA
15	76602360	76602415	ETFA
15	80444822	80444897	FAH
15	80445112	80445291	FAH
15	80445294	80445297	FAH
15	80448283	80448425	FAH
15	80448429	80448432	FAH
16	30999652	30999653	HSD3B7
16	30999660	30999939	HSD3B7
16	71601098	71601257	TAT
16	71611002	71611003	TAT
17	3564838	3564838	CTNS
17	3565388	3565535	CTNS
17	3565606	3565606	CTNS
17	7121025	7121027	ACADVL
17	7127900	7127900	ACADVL
17	7127905	7127966	ACADVL
17	26727722	26727722	SLC46A1
17	41064988	41064995	G6PC
17	73747665	73747748	GALK1
17	73759039	73759039	GALK1
19	1398155	1398594	GAMT
19	1401564	1401579	GAMT
19	13003070	13003107	GCDH
19	13003121	13003258	GCDH
19	40736733	40736906	AKT2
19	40743339	40743473	AKT2
19	40748831	40749009	AKT2

19	40749692	40749933	AKT2
19	40749964	40749966	AKT2
19	40774718	40775002	AKT2
19	40790946	40791009	AKT2
19	40791078	40791312	AKT2
19	41903574	41903574	BCKDHA
19	41924620	41924669	BCKDHA
19	45409607	45409614	APOE
19	45410124	45410360	APOE
19	45411199	45411219	APOE
19	45411780	45411783	APOE
19	45452269	45452269	APOC2
19	45452271	45452320	APOC2
19	45452327	45452329	APOC2
19	45452334	45452336	APOC2
19	51849086	51849189	ETFB
19	51851481	51851482	ETFB
19	51851489	51851505	ETFB
19	51851550	51851558	ETFB
19	51851563	51851642	ETFB
19	51858400	51859327	ETFB
19	51869595	51869682	ETFB
20	746424	746432	SLC52A3
20	746741	746742	SLC52A3
20	746764	746768	SLC52A3
20	746772	746798	SLC52A3
20	32889903	32890008	AHCY
20	32890011	32890013	AHCY
20	32890015	32890015	AHCY
20	32890017	32890018	AHCY
20	32890020	32890023	AHCY
20	43280384	43280409	ADA
21	38121747	38121747	HLCS
21	38302544	38302544	HLCS
21	38320323	38320597	HLCS
21	38338734	38338979	HLCS
21	44495977	44496065	CBS
21	44496475	44496488	CBS
21	44496491	44496512	CBS
22	51062090	51062394	ARSA
22	51062562	51062562	ARSA
22	51062565	51062753	ARSA
22	51063152	51063170	ARSA
22	51066570	51066617	ARSA
X	152991611	152991631	ABCD1

WES

**Gene**	**Genomic location (GRCh37)**	**GENCODE transcript**	**Exon location in transcript**
ARG1	Chr6:131,868,263-131,868,338	ENST00000672233.1_1	Exon 2 (from start) out of 8
BTD	Chr3:15,709,536-15,709,748	ENST00000673467.1_3	Exon 4 (till stop) out of 4

BTD	Chr3:15,713,117-15,713,140	ENST00000673620.1_5	Exon 4 (till stop) out of 4
BTD	Chr3:15,751,567-15,751,581	ENST00000672141.1_5	Exon 4 (till stop) out of 4
BTD	Chr3:15,751,906-15,751,932	ENST00000672760.1_3	Exon 4 (till stop) out of 4
BTD	Chr3:15,763,277-15,763,356	ENST00000672427.1_4	Exon 5 (till stop) out of 5
CA5A	Chr16:87,915,417-87,915,545	ENST00000649158.1_3	Exon 7 (till stop) out of 7
CTPS1	Chr1:41,469,653-41,469,786	ENST00000463285.7_3	Exon 13 (till stop) out of 13
FBP1	Chr9:97,400,625-97,400,692	ENST00000648117.1_3	Exon 1 (from start) out of 6
MMADHC	Chr2:150,428,744-150,428,845	ENST00000422782.2_3	Exon 7 out of 9
PGM1	Chr1:64,120,661-64,120,782	ENST00000650546.1_1	Exon 11 out of 12
SLC19A3	Chr2:228,550,866-228,550,885	ENST00000409287.5_3	Exon 4 (till stop) out of 4

WGS

Two genes with low coverage in males

ABCD1	chrX: 152,990,323-153,010,216 forward strand
OTC	chrX: 38,211,798-38,280,703 forward strand

One gene with low coverage overall in both sexes (due to pseudogene)

CBS	chr21: 44,473,301-44,497,053 reverse strand
